# Rethinking science-policy-practice interaction for transformative sustainability research and innovation in Europe

**DOI:** 10.12688/openreseurope.21755.2

**Published:** 2026-01-14

**Authors:** Jari Lyytimäki, Leena Kunttu, Stephan Bartke, Karl Henry Eckert, Gerald Jan Ellen, Helmut Gaugitsch, Boris Lipták, Linda Maring, Erkki Mervaala, Camilo Molina, Judith Neumann, Sonja Otto, Bart Rijken, Kester Savage, Mariësse van Sluisveld

**Affiliations:** 1 Finnish Environment Institute, Helsinki, Uusimaa, Finland; 2 Umweltbundesamt, Dessau-Roßlau, Saxony-Anhalt, Germany; 3 Deltares, Delft, South Holland, The Netherlands; 4 Environment Agency Austria, Wien, Austria; 5 Slovak Environment Agency, Banská Bystrica, Slovakia; 6 Natural Resources Wales, Lowestoft, UK; 7 PBL Netherlands Environmental Assessment Agency, The Hague, The Netherlands

**Keywords:** Communication, Innovation, Interaction, Science, Sustainability, Participation, Policy, Transformation

## Abstract

Impactful knowledge generation and utilisation, aiming at addressing environmental and sustainability challenges, requires meaningful interaction between scientists, stakeholders, policymakers and society. This study identifies key success factors and challenges at the science-policy-practice interaction, drawing on document analysis, interviews, and workshops involving experts, policymakers, and funders of environmental and sustainability research and innovation across 14 European countries. Experiences from current practices highlight the highly variable contexts for interaction and communication throughout Europe, a diverse range of tools and approaches, and differing levels of available resources. Siloed structures and the need to engage various societal sectors and levels of governance remain significant challenges. The development and employment of expertise in communication, interaction and knowledge co-creation, capable to orchestrate this variability, is essential. Greater recognition of the diverse dimensions of inclusive participation is proposed to ensure that environmental and sustainability research and innovation, aimed at societal transformation, leaves no one behind.

## Plain Language Summary

Sustainability science is only useful if it reaches the people who make decisions about climate, ecosystems, or resources. The present study therefore set out to investigate how scientists, policymakers, and society actually interact in Europe—a continent remarkably diverse in terms of politics, culture, and resources—and what makes these interactions succeed or fail. The work draws on desk reviews, semi-structured interviews with approximately 300 experts (researchers, politicians, and funding bodies) across fourteen European countries, and workshops where participants tested the findings. The aim was to learn from real-world situations: the communication tools used, their funding mechanisms, and the practices and structures that facilitate or hinder dialogue. A key claim is that without inclusive, broad-based dialogue, European sustainability science risks remaining a collection of isolated projects rather than a coordinated engine for societal transformation.

## Introduction

Communication and interaction are essential for efficient knowledge generation and utilisation, which are at the heart of the sustainability transition. Various assessments and scientific studies have shown that the evidence base for decision-making is already sufficient to justify rapid and large-scale transformative action (e.g. EEA, 2019; EEA, 2025; Kallis *et al.*, 2025). However, this evidence has not yet translated into actionable change capable of slowing down or even eradicating unsustainable pressures on climate ( IPCC, 2023), biodiversity ( IPBES, 2024), or resource use ( UNEP, 2024).

In other words, the great acceleration of environmental pressures continues despite science-based calls for sustainability transition ( Steffen *et al.*, 2015). These calls have been made repeatedly in various forms, from the 1972 United Nations Conference on the Human Environment in Stockholm to the 1992 Earth Summit in Rio de Janeiro and from the eight Millennium Development Goals targeting 2015 to the seventeen Sustainable Development Goals (SDGs) targeting 2030. These calls and processes have gained episodic media attention, influencing public and policy agendas ( EEA, 2021; Holt & Barkemeyer, 2012). This, in turn, has partly shaped priorities in research and innovation (R&I) activities in global, European and national levels. However, the impacts have been insubstantial. For example, the SDGs have influenced societal development primarily on a discursive level by repackaging existing initiatives using the language of the SDGs, rather than initiating transformative action ( Biermann *et al.*, 2022; Lyytimäki *et al.*, 2025; UN, 2025).

Addressing sustainability transitions requires navigating a polycrisis of climate change, biodiversity and habitat loss and the impact of environmental pollution on human and ecosystem health as well as crises in the political, economic and social spheres, the 'slowbalisation' of the global economy and trade and growing societal fragmentation over values and identities ( EEA, 2023). Misinformation and disinformation, exacerbated by social media, as well as the rise of populism and outright science denial, are challenges for communicating this evidence to decision-makers ( Ekberg *et al.*, 2022). Rapidly evolving communication and interaction applications employing machine learning and artificial intelligence bring in new possibilities but also unforeseeable risks. Despite the increasing knowledge base on environmental and sustainability issues, prospects for coherent and forward-looking evidence-based decision-making might be eroding in the 2020s. The tension between the abundance of knowledge and the lack of political and societal uptake has been described as the ‘knowledge–action gap’ ( Räsänen *et al.*, 2024). Addressing this gap requires moving beyond evidence accumulation towards processes that co-produce actionable knowledge.

Here we review recent experiences of science-policy-practice-interaction (SPPI) across Europe and call for a genuinely inclusive and just approach to ensure that sustainability research and innovation benefits all. Our primary aim is to map and synthesize the approaches, tools and methods currently employed for environmental and sustainability SPPI, and to formulate recommendations for enhancing their effectiveness. We focus on Europe but aim to draw lessons that are widely applicable across different regions and topics of communication. This study is motivated by sustainability transition literature ( Köhler *et al.*, 2019; Vähäkari *et al.*, 2020) and rooted in the literatures of science communication and interaction. Communication and interaction have received relatively little attention in transition studies despite being at the heart of transition processes such as innovation diffusion and knowledge utilisation. However, they offer valuable insights and lessons relevant to sustainability transition. Relevant areas include sub-fields of science communication such as climate communication ( Boykoff, 2011) and sustainability communication ( Godemann & Michelsen, 2011), research on science advice and knowledge brokerage ( Gluckman *et al.*, 2021), knowledge co-creation and citizen engagement ( Huttunen *et al.*, 2022), and risk communication and governance ( Renn, 2008). We draw from these diverse areas to explore the role of communication and interaction within the context of national and European R&I systems in relation to sustainability.

Our main claim is that a more inclusive approach to science-policy practice interaction is needed to make knowledge generation and interaction effective and capable of fully supporting sustainability transformation. We justify the claim based on European examples and experiences of SPPI. The next section describes the materials we draw upon.

## Background and methods

We use insights from document analysis, interviews, and workshops with experts in environmental and sustainability research and innovation across Europe. The focus is on practical experiences and concrete examples. The materials were collected under the EU funded Coordination and Support Action “Collaborative Action coordinating and enhancing systemic, actionable and transversal Sustainability Research and Innovation” (CASRI, www.casri.eu). One of the main aims of the project is to identify good practices and gaps in SPPI across Europe. This aligns with the project’s overall goal to outline a strategic European R&I agenda that stimulates collaboration on key topics for environmental and sustainability R&I and thereby promoting sustainable development in Europe in times of multiple crises.

This study draws on academic literature, desk studies and expert stakeholder views from fourteen European countries and regions, including Austria, Basque Country (Spain), Bulgaria, Flanders (Belgium), Finland, France, Germany, Ireland, Italy, Montenegro, The Netherlands, Slovakia, Switzerland and Wales (United Kingdom). A three-step procedure was developed. First, in each country or region, national contact persons familiar with the local context identified a pool of relevant national key stakeholders. The goal was to assemble diverse national samples of senior experts, representing both the private and public sectors and possessing expertise across a range of sustainability R&I fields, with balanced gender representation ( Bartke, 2024). More specifically, stakeholders were expected to have expertise on at least one of the project’s key themes: circular economy, biodiversity and climate, sustainable urbanisation, and energy transition.

**Figure 1.**
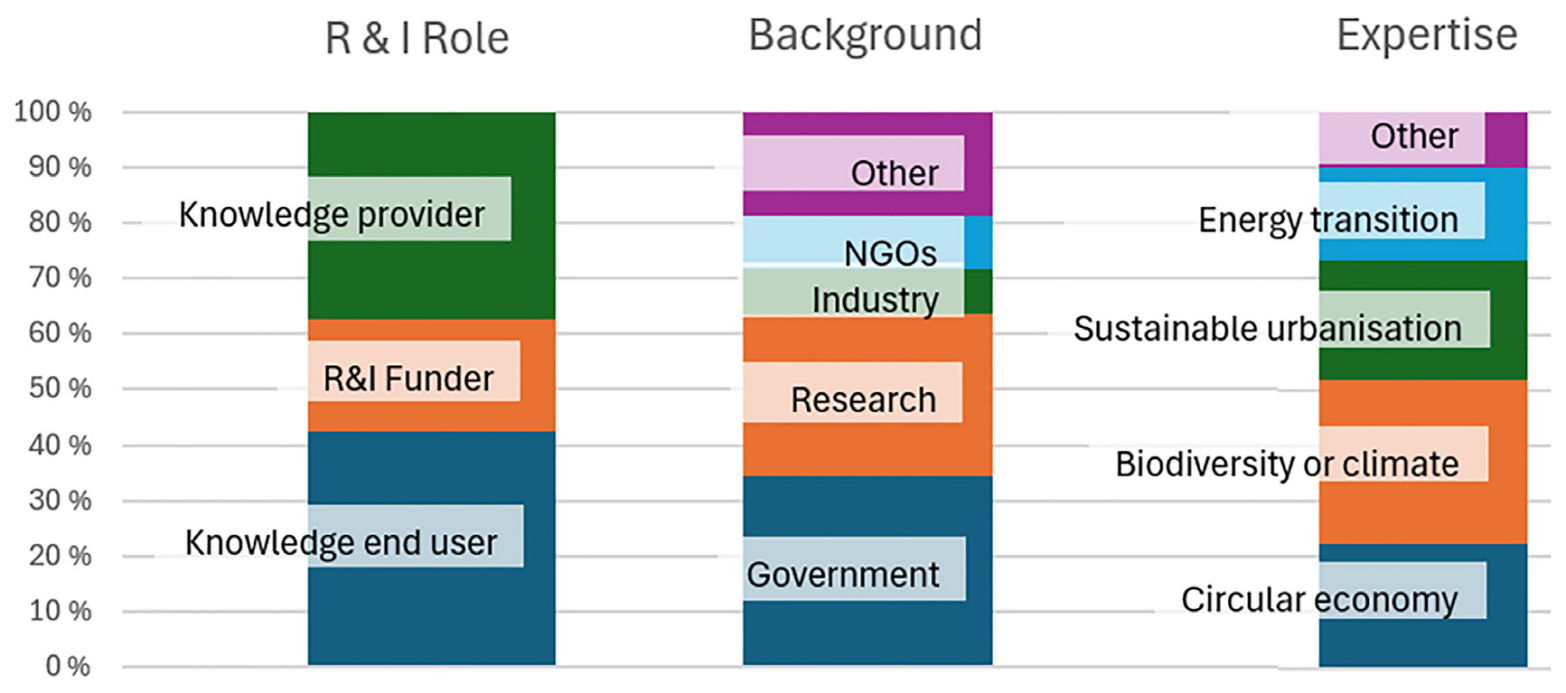
Main roles in research and innovation (R &I) processes, organizational backgrounds and key areas of expertise of the respondents (adapted from Maring *et al.*, 2025). Note:, a single respondent can have multiple roles and affiliations or expertise on multiple themes.

Second, national desk studies on the project’s key themes were performed to generate up-to-date national overviews. These studies reviewed key R&I agendas, scanned national funding schemes, and examined relevant aspects of SPPI. This information supported the interviews, which served as the primary source of data. In each country, approximately 20 relevant expert stakeholders were interviewed. Of the interviewees, 43% were female and 57% male. Figure 1 summarises the background of the interviewees.

A semi-structured interview scheme, including detailed instructions to guide the interviews, was prepared ( Maring *et al.*, 2025, Appendix C). Following this guidance, all interviews were conducted in national languages and subsequently reported in English. Interviews were conducted during spring and summer 2024. Most interviews were conducted in person; recordings were obtained for nearly all sessions. The interview scheme comprised three main sections: R&I priorities, funding opportunities, and SPPI. The SPPI section included three key questions:

- Please describe what the interaction between science, policy, and practice looks like in your work?
- Please describe what activities and/or organisations in your field of expertise effectively facilitate the interaction between science, policy, and practice? How are you involved in these?
- What are the main challenges for this/these activities and/or organisations at the moment? What can be reinforced or improved? Or should other science policy structures and activities be developed?

An optional question for respondents with significant experience in SPPI was included, aiming to gain a deeper understanding of the capacities needed to improve interaction. Topp *et al.*’s (2018) typology of interaction capacities provided a framework for considering key needs and types of organizations, both nationally and internationally. However, due to time constraints and the breadth of topics covered, none of the fourteen cases was able to fully address the typology during the interviews.

The third step involved initial analysis, national stakeholder workshops designed to review, synthesize, and prioritise the gathered information, and compilation of the results into a single document. Workshops followed a shared general template, which was adapted to national specificities ( Maring *et al.*, 2025, Appendix D). Participants were selected from the interviewees and, on occasion, included additional key stakeholders. The workshops provided participants with an opportunity to summarise, review and reflect on the views of others and highlight the most important issues.

Small teams of nationally based researchers were responsible for initial analysis, organization of interviews and workshops, and data analysis. Recognising the high diversity of national contexts, participant backgrounds, and topics addressed, the analysis relied on qualitative interpretations and aimed to summarize key points related to SPPI for international comparison, following a comparative case study approach ( Bartlett & Vavrus, 2016). Cross-country coherence of interpretations was ensured through multiple rounds of review by project coordination, designated theme leaders, and the project’s international advisory board. The results were compiled into national comprehensive reviews and synthesised into a single, openly available report, which serves as material for this analysis ( Maring *et al.*, 2025). Participants had a final opportunity to check the content of the national reviews via a survey. Content-based errors identified by them were corrected and their further recommendations were documented as an appendix to the final report.

Small teams of nationally based researchers were responsible for initial analysis, organization of interviews and workshops, and data analysis. Recognizing the high diversity of national contexts, participant backgrounds, and topics addressed, the analysis relied on qualitative interpretations and aimed to summarize key points for international comparison, following a comparative case study approach ( Bartlett & Vavrus, 2016). Cross-country coherence of interpretations was ensured through multiple rounds of review by project coordination, designated theme leaders, and the project’s international advisory board. The results were compiled into national comprehensive reviews and synthesized into a single, openly available report, which served as the basis for this analysis ( Maring *et al.*, 2025). Participants had a final opportunity to review the content of the national reviews via a survey. Content-based errors identified by them were corrected, and their further recommendations were documented as an appendix to the final report.

While the approach did not constitute a full-scale transdisciplinary co-production process, it sought to capture multiple perspectives and highlighted potential tensions between knowledge supply and demand. The data collection integrated broad bottom-up stakeholder engagement and iterative synthesis, balancing national diversity with transnational coherence ( Firus *et al.*, 2024). Building from earlier experiences ( Bartke *et al.*, 2018), this flexible yet structured method aimed to ensure inclusivity while resulting in actionable knowledge. However, several limitations of the method should be acknowledged. Due to resource constraints, the number of participants was kept relatively low, potentially limiting the breadth of expertise and increasing the risk of uneven thematic or sectoral representation within the national cases. The time allocated for discussing SPPI in the interviews and workshops was limited, which may have resulted in omissions or superficial treatments of certain issues. Variations in the expertise and disciplinary backgrounds of the national teams conducting the initial analysis introduced potential risk for interpretative differences. Consequently, this study relies on qualitative interpretation and iterative consensus building and validation among authors. Furthermore, the primary data for this analysis are drawn from the findings documented in Maring *et al.* (2025), without conducting further analysis of the underlying data, which are reported in national languages. Translation from national languages to English also presented a potential source of error; national language executive summaries were provided with each final national report in English to ensure accurate conveyance of key messages. Finally, while the results reflect a variety of contexts, they are inherently limited by a focus on European perspectives, primarily representing high-income European contexts.

This study is structured as follows. The following section summarises the results, presents illustrative examples and discusses the success factors and challenges of SPPI in Europe. Based on qualitative content analysis, we highlight (1) approaches adopted, (2) resources and their use, (3) organisational structures, (4) policy contexts, and (5) inter- and transdisciplinary practices. The results are then examined through the lens of inclusive participation, highlighting the need for a more comprehensive understanding of inclusiveness within interaction and communication processes ( Kalliomäki *et al.*, 2024; Kalliomäki *et al.*, 2025; Kunttu & Lyytimäki, 2025; Lawson, 2010; Moulaert *et al.*, 2013). Using relational trust building, equitable participation, and inclusiveness as key criteria, we propose practical steps for SPPI supporting sustainability transition. Finally, concluding recommendations are provided to support the formulation and implementation of a transnational strategic research and innovation agenda designed to support SPPI capable of addressing key sustainability challenges and opportunities. Key criteria for outlining the recommendations based on the lessons learned from the European cases include long-term societal impact of SPPI, capability to address multi-faceted challenges, and effectiveness on targeting and engaging different target groups. These recommendations are guided by the lessons learned from our European cases, emphasizing the long-term societal impact of SPPI, the ability to address multifaceted challenges, and the effectiveness of targeting and engaging diverse stakeholder groups.

## Results and findings: Identifying key European SPPI challenges

### A variety of tools and SPPI approaches already in use

Together with earlier studies ( Davies *et al.*, 2021; Gluckman *et al.*, 2021; Lyytimäki *et al.*, 2013; Topp *et al.*, 2018; Trench & Miller, 2012), our cases demonstrate a wide range of communication and interaction approaches and methods are already being utilised in Europe ( Table 1). Variations were found between countries, reflecting differing socio-cultural contexts, policy systems, economic conditions, geographical settings, and institutional arrangements. Differences also appear within individual countries, where some policy domains rely on traditional communicative tools and well-established practices, while others are actively experimenting and adapting to social and technological developments. Table 1 outlines a synthesis of the key elements of SPPI presenting examples provided by the respondents through simplified dichotomous continuums.

**Table 1.** European examples of different SPPI approaches and tools already operational (Data source: Maring *et al.*, 2025).

Key elements	Continuums of types of activities	Examples mentioned by the respondents
Information sharing	One-way tools for informing **↕** Two-way approaches for co- creation	Policy briefs; Websites; Open databases; Newsletters; Press releases **↕** Knowledge co-design workshops; Participatory citizen assemblages
Science advice mechanisms	Permanent science advice bodies **↕** Ad-hoc or temporary activities	Advisory councils; Science panels; Innovation policy coordination offices; Regional science hubs **↕** Events and campaigns; Science festivals; Thematic days; Awards
Context-specificity	Highly personalised and targeted approaches **↕** Generic communication for wide audiences	Science advisers and ambassadors, One-to-one discussions, Education and training of researchers and policy makers, Policy co-design workshops **↕** Media coverage and interviews, Websites
Science-policy emphasis	Research-driven approaches **↕** Policy-driven approaches	Permanent research centres and technology transfer offices, National institutes **↕** Consultants, Public relations firms, Governmental science advisors or units, Science sparring processes
ES R&I steering influencing SPPI	Specific and formal governmental policies **↕** Generic societal pressures	National and EU level strategies, Government Science-Technology- Innovation (STI) programmes, Official policies, Mission statements **↕** Stakeholder pressure, Public interest, Expectations for economic, environmental or social gains

Various national information sharing platforms facilitate collaboration between scientists, policymakers, and practitioners. Science-driven information sharing was frequently reported and increased activity within the communication departments of research institutions and universities was observed. These efforts often represent science public relations—a traditional, one-way dissemination strategy aimed at presenting and popularising research results. In a contested communication environment this approach faces increasing risks, particularly concerning trust-building between different actors ( Weingart & Guenther, 2016). In some cases, communication and interaction were integrated from the outset of the research process to foster genuine engagement and more effective outreach. For example, the Strategic Research Council of Finland requires funding applicants for multi-year, cross-disciplinary research consortiums to present a detailed communication and interaction plan. The Council also encourages stakeholder interaction already during the initial outlining of research tasks.

Well-established science advice mechanisms include advisory councils or dedicated offices or units within government bodies, responsible for summarising, analysing, and presenting evidence tailored to policy needs. National science panels, composed of prominent scholars, were also noted by respondents. These bodies are typically mandated to operate independently, as their credibility relies on their ability to provide impartial advice to policymakers. Some evidence of collaboration between national science panels and international panels, such as the Intergovernmental Panel on Climate Change (IPCC) and Intergovernmental Science-Policy Platform on Biodiversity and Ecosystem Services (IPBES), emerged. Collaboration between science panels at the European level was not mentioned, despite the existence of networks designed to support such collaboration, such as the European Environment and Sustainable Development Advisory Councils Network (EEAC). Consequently, there appears to be an opportunity to mobilise existing actors through a ‘network-of-networks’ approach ( Kelemen *et al.*, 2021).

Context specific instruments designed to facilitate learning through dialogue include various networks, individual actors, and institutions. An example from Flanders is the 'science ambassador' role, which includes a dedicated training programme focusing on the circular economy. Thematic multistakeholder networks or platforms were frequently highlighted, particularly from a technology and business standpoint. For example, triple or quadruple helix spearhead clusters for innovation have been proposed as approaches suitable for more wide-ranging SPPI. These approaches aim to foster dialogue between industry, knowledge providers, authorities, policymakers, and societal stakeholders, including non-governmental organisations ( González-Martínez *et al.*, 2025). Even unidirectional communication tools can be used to initiate interactive and co-creative dialogues. An example from Switzerland demonstrated how multiple non-governmental organisations can be involved to develop a policy brief, while an example from Finland highlighted the use of a concise science-based policy brief to stimulate interest among key policymakers, which led to in-depth face-to-face discussions on the recommendations presented.

Media and social media were considered both factors within the context of SPPI and tools for its implementation. The potentially divisive and polarising nature of social media was noted, alongside its potential for information sharing and targeted discussions. Press releases and media coverage are important for information sharing and public awareness, but the role of media and journalists was mentioned only occasionally. It was noteworthy that science journalism was not frequently mentioned, despite its traditionally important role in shaping the environmental and sustainability agenda ( Godemann & Michelsen, 2011; Sachsman & Myer Valenti, 2020).

SPPI can also be outsourced to consultants, public relations firms, or knowledge brokers. Concerns were raised about the use of consultants in some countries (France, Slovakia), as this practice can create vulnerabilities to lobbying influence, lead to the outsourcing of knowledge generation and diminish understanding of the issues among administration and decision-makers.

Developing interpersonal skills and sustaining motivation for interaction emerged as important factors from multiple perspectives. Depending on the context, communication based on personal relationships was viewed either as a means for effective interaction (Germany, Switzerland) or as an indication of dysfunctional institutional communication (Slovakia). Researchers often lack incentives for outreach, and policymakers often face time and capacity constraints in addressing complex sustainability issues. A lack of science literacy also emerged as a key concern, particularly for complex issues with relatively low public and policy attention, as demonstrated by the emphasis on 'soil literacy' as one of the main objectives of the European mission on soil ( EC, 2021).

Concerns were raised about disinterest and even reluctance to adopt science-based recommendations among some policymakers. This can be linked to a lack of public trust in science in some cases. In France, disinterest or distrust towards environmental and sustainability studies among certain end-users was noted. Targeted training and education to support systematic collaboration were suggested as beneficial, particularly in fostering collaboration and building trust between (early career) researchers and policymakers.

### Availability and coordination of resources for SPPI

The level of funding and resources available, the degree of institutional support and coordination, and the perceived effectiveness of current SPPI practices varied considerably. Countries such as Switzerland, Wales, and Ireland exhibited comparatively established practices for communicating with policymakers across a range of channels. In France, research and SPPI benefits from considerable government funding. Flanders also demonstrated a robust SPPI framework, with numerous concertation structures, information platforms, research initiatives, and related events. In Ireland, a significant number of government agencies and departments offer competitive R&I funding in the areas of climate, the environment, and sustainability.

Conversely, insufficient or even lacking funding for SPPI remains a significant challenge, particularly in smaller countries with fewer resources. National funding schemes in Slovakia are relatively small compared to EU and international initiatives. Bulgaria faces primary challenges in developing research infrastructure. Consequently, SPPI may not be prioritised within the research process.

However, even when resources are available, the use of those resources and competencies for coordination may present a more pressing issue ( Juhász *et al.*, 2024; Kelemen *et al.*, 2021). In some cases, limitations with the rules and formats of funding can hinder efficient SPPI. While some countries possess robust funding systems, inefficiencies, bureaucratic processes, and complex regulations can impede the effective use of available resources. In Switzerland, a key challenge lies in strategically aligning different funding instruments. The issue is not necessarily a lack of funding, but rather a duplication or even muddling of efforts across specific areas.

Respondents frequently highlighted a lack of long-term, dedicated funding for SPPI. Funding often focuses on specific, narrowly defined areas, or is provided sporadically for short-term projects or experiments. These funding structures often determine the degree to which actionable science can be produced, echoing recent findings that long-term, flexible funding is crucial to bridge the knowledge–action gap ( Nyboer *et al.*, 2021). Therefore, this poses a particular challenge for SPPI addressing broad, long-term environmental protection and sustainability transformation. Boundary organizations, which facilitate knowledge exchange between science and policy, were particularly identified as underfunded. Although R&I should remain politically independent, political support and commitment are very important, to ensure sufficient attention for SPPI. This demonstrates how vulnerable SPPI can be to political shifts, as evidenced by Finland’s experience in 2023, when the right-wing government abolished its system for inter-ministerial analysis, assessment, and research activities.

Potential avenues for supplementing inadequate national public funding included public-private partnerships and crowdfunding. However, these approaches present challenges in smaller market areas with limited populations and risk SPPI becoming overly influenced by commercial interests and focusing on issues with high public appeal. EU funding and international grants were also considered potential sources of additional funding for SPPI.

### Siloed structures and compartmentalization hinder SPPI

Fragmented organizational structures and a project-based working culture were identified as significant barriers to effective SPPI. Funding schemes and SPPI practices are frequently compartmentalized within siloed structures. As noted by earlier research ( Munck af Rosenschöld, 2023), this may result in individual projects that often fail to encompass the broader scope of sustainability. Such structures and the fragmented governance landscape remain significant challenges but also reveal spaces where institutional entrepreneurs within the public sector can mobilise networks and lead by example ( Alexandrescu *et al.*, 2014). Yet, overly strict compartmentalization within science can lead to inefficient resource allocation in SPPI, potential knowledge gaps, and a limited exchange with policymakers. Similarly, compartmentalized structures within government agencies were observed to impede collaboration with the scientific community, highlighting the importance of increased dialogue. Finland and the Netherlands were cited as examples of countries with relatively flat hierarchies, which can facilitate communication across administrative silos.

SPPI tools and initiatives are often implemented temporarily as part of short-term projects, dependent on fluctuating funding availability, public interest, or shifting policy priorities. This short-term focus introduces discontinuity and imposes time constraints on collaboration across all levels, from knowledge generators to end-users. Rapidly evolving R&I priorities, combined with the demands of media and social media, place considerable pressure on the speed of knowledge dissemination, exacerbating existing time limitations. This can also detract from the development of a fundamental and strategic knowledge base as resources are directed towards addressing immediate concerns. These factors contribute to difficulties in achieving coherent SPPI and underscore the need for improved coordination among ongoing projects.

Respondents from multiple countries observed that fragmented organizational structures and divergent administrative practices result in inefficient resource allocation. Facilitating personnel transfers between sectors could contribute to improved integration. Initiatives that actively convene researchers, businesses, and policymakers are essential. The establishment of boundary organizations or knowledge ecosystems to connect projects and overcome siloed approaches was consistently highlighted. Ultimately, respondents stressed the necessity for enhanced coordination of R&I initiatives to generate more systemic knowledge and drive transformative change. For example, Slovakia underscored the importance of cross-disciplinary research approaches. Findings and methodologies from one research area should be systematically applied to others.

### Different policy contexts for SPPI

Ensuring that research outcomes are readily applicable and genuinely useful within specific local contexts was emphasised as a basic function for SPPI. Current capacities to utilise SPPI differ considerably. As highlighted in Flanders, SPPI addresses a diverse spectrum of needs among various stakeholders, encompassing policy support, technological development, heightened citizen awareness, societal acceptance, resources for demonstration and implementation, robust monitoring methodologies, and a deeper understanding of human behaviour. Conversely, in Italy, research outcomes remain underutilised, risking remaining purely theoretical. A significant challenge in Italy is the difficulty faced by the nation’s extensive network of small and medium-sized enterprises (SMEs)—a defining feature of the productive sector—in accessing R&I, hindering their progress towards green, energy, digital, and technological transitions. Similarly, in Austria the need to redefine the role of SMEs within R&I activities was highlighted.

The diversity of national contexts and policy systems influencing SPPI presents a significant obstacle to the development of recommendations applicable across Europe. Overly centralised systems can impede effective engagement by failing to account for local contexts and regional requirements, as observed in France. The federal structure of Germany was noted as a source of complexity in coordinating diverse regional interests and ensuring equitable representation. Regional and linguistic diversity complicates policy coordination and communication, as evidenced by the experience of the Basque Country. Bureaucratic processes can impede the translation of scientific research into actionable policies, thereby affecting implementation, as highlighted by observations from Italy.

The diversity of policy contexts also presents unique national approaches for SPPI. For example, Wales possesses a distinct legislative framework through its Well-being of Future Generations Act (2015) and Environment (Wales) Act (2016), the first of their kind to legally incorporate the UN Sustainable Development Goals. This framework mandates a joined-up approach across the public sector and requires the Sustainable Management of Natural Resources, thereby embedding an integrated consideration of environmental, social, and economic well-being into all levels of policy and practice. This provides a strong, legally-backed context for SPPI that differs significantly from other regions.

The significance of established knowledge producers—including universities, research institutions, governmental advisory bodies, and expert councils—was consistently highlighted. These organizations typically provide expert knowledge to inform policy formulation. The influence of such bodies is contingent upon the prevailing policy environment. In Ireland, the Environmental Protection Agency (EPA) Research Programme coordinates national environmental research initiatives through the National Environmental Research Coordination Group. Every three years, the EPA conducts a comprehensive assessment to determine thematic research priorities. This process facilitates the long-term strategic development of SPPI by ensuring alignment between research and policy objectives.

However, established knowledge infrastructures also present a risk of exacerbating bureaucracy and administrative complexity, potentially hindering access to funding, project implementation, and effective communication. This risk was noted in relation to both European-level funding and within countries like Italy. Therefore, ensuring streamlined and accessible processes is vital, particularly for new entrants to SPPI, to foster broad and effective participation.

### The challenge of transformative transdisciplinarity

Across the surveyed countries, the need for more diverse and interactive communication to foster transformative collaboration—research intrinsically linked to societal change—at local, national, and international levels was frequently emphasized. In Switzerland, it was observed that funding mechanisms should be better aligned with transformative research by fostering unconventional forms of scientific collaboration, experimentation, and outputs. However, the continued need for conventional, unidirectional science communication was also acknowledged. This underscores the necessity to utilize the diverse tools of SPPI through the integration of complementary approaches.

Addressing complex sustainability challenges undeniably requires the inclusion of diverse fields of expertise ( Godemann & Michelsen, 2011). However, effectively bringing these disciplines together necessitates significant effort in cultivating mutual understanding and establishing effective modes of collaboration between fields of natural sciences, the humanities and social sciences, each with its own inherent culture and methodologies. Beyond interdisciplinarity, there is a growing need for transdisciplinary collaboration, which explicitly involves non-scientific actors, such as citizens, policymakers, and professionals from the productive and creative sector, media and arts.

To facilitate this, emphasis was placed on creating supportive environments that encourage dialogue and collaboration. The practical challenges associated with transdisciplinary research, particularly those involving non-scientific actors, are, however, pronounced ( D'Amato *et al.*, 2025). For instance, in Italy, improved coordination of funding mechanisms alongside this dialogue was deemed essential. Similarly, in France and Austria, fostering transdisciplinarity and the collaborative construction of research with diverse actors—including citizens, associations, firms, and local authorities—was recognised as vital. Furthering this, Wales is actively engaged with international forums such as the UNESCO-Bridges Coalition, which serves as an exemplar for foregrounding the humanities and social sciences in developing solutions to the climate and nature crises, reinforcing the need for truly transdisciplinary approaches.

## Towards more inclusive SPPI

A recurring theme emerging from the European experiences was the need for more broad-based and inclusive SPPI, facilitating a just transition towards sustainability. This isn’t just a matter of fairness; it is essential for ensuring that transition efforts are effective, acceptable and sustainable for all. Achieving inclusivity requires specific practices and policies designed to dismantle barriers to participation amongst under-represented individuals, social groups, firms, sectors, and regions, while fostering the integration of sustainability research and entrepreneurial activities. Ultimately, the objective is to ensure equitable opportunities for all segments of society to engage with and benefit from environmental and sustainability R&I ( Lähteenmäki-Smith *et al.*, 2023; Planes-Satorra & Paunov, 2017). Collaborative co-creation and co-design SPPI processes exemplify this inclusive approach, involving diverse stakeholder groups in initiatives aligned with shared interests, such as those related to urban planning tasks ( Lee *et al.*, 2024) or in developing urban transformative capacity ( Wolfram, 2016).

Inclusion has already been recognised as a critical dimension of R&I policy, contributing to greater equitability and sustainability by fostering the acceptance of policy missions and facilitating their implementation ( UN, 2023). Effective policy action necessitates a comprehensive understanding of its potential impacts across diverse stakeholder groups, ensuring the involvement of all relevant parties throughout planning, implementation, monitoring and evaluation. It is crucial to acknowledge that incorporating all stakeholders into the decision-making process can present challenges and risks ( Bogner & Dahlke, 2022). These include the possibility of premature assumptions for consensus, the risk of dominant actors unduly influencing outcomes, and the adoption of overly simplistic solutions that prove difficult to scale ( Hermans *et al.*, 2011).

Therefore, careful consideration must be given to involving relevant and representative stakeholder groups and managing the process in a structured and systematic manner ( Firus *et al.*, 2024). A balanced approach is needed—one that actively seeks diverse voices while remaining alert against potential pitfalls and ensuring that outcomes remain grounded in robust evidence and a clear understanding of the complexities of the transition process. Fundamentally, inclusive SPPI represents a process of fostering trust, with the assurance that equitable solutions addressing the divergent impacts of the transition process will be identified, even in a context of long-term, global environmental change.

To further assist in achieving this, Box 1. presents practical steps towards more inclusive SPPI, grounded in the principles of relational trust building, equitable participation and inclusiveness. These steps summarise key lessons from our empirical European cases and are designed to serve as an accessible and actionable blueprint adaptable to various communication contexts, with a focus on fostering lasting, impactful change towards sustainability.

**Box 1. Practical steps towards inclusive SPPI supporting sustainability transition**

**Institutionalise SPPI platforms for trust-building**

Establish dedicated, multi-stakeholder 'Sustainability SPPI Forums' at national and sub-national levels. These Forums should prioritise the representation of marginalized communities and individuals directly impacted by sustainability transitions. Design these forums with a deliberate focus on fostering mutual understanding and trust, embedding structured facilitation methods, participatory narrative-building and transparent decision-making rules to bridge divergent perspectives and prevent elite capture. Implement independent regular monitoring and evaluation of participation and equity outcomes to ensure inclusivity and adapt Forum structures as needed.

**Reframe solutions to prioritise empowerment**

Organise collaborative 'Problem Definition Workshops' co-led by scientists, policymakers, and community representatives. These workshops should explicitly examine underlying power dynamics and systemic inequalities that contribute to environmental and social challenges. Frame solutions not as technical fixes or top-down regulations, but as collaboratively designed interventions that address root causes and empower communities to become active agents of change.

**Increase societal relevance through localised expertise**

Develop a 'Contextualization Toolkit' for SPPI, including adaptable communication strategies, facilitation techniques, and resource allocation models. This toolkit should be designed to guide practitioners in tailoring SPPI tools to specific cultural, economic, and political contexts while ensuring alignment with EU and global objectives. Invest in localised long-term, independent knowledge brokers who possess the linguistic and cultural competency and legitimacy within their communities to facilitate effective communication and build trust within diverse communities.

**Address cross-systemic knowledge needs**

Establish 'SPPI Innovation Networks' at the intersection of academic institutions, government agencies, and civil society organizations. These networks should be staffed by individuals trained in systems thinking, facilitation, and relational leadership – to identify leverage points for change, build cross-sectoral partnerships, and foster collaborative problem-solving. Mandate cross-sectoral rotations for staff within these networks and introduce joint training on systems thinking and relational leadership to break down internal silos and build a shared culture of collaboration.

**Address coordination and funding needs**

Create a 'SPPI Coordination Fund' designed to provide long-term financial and administrative support. This fund should prioritize actors and networks demonstrating commitment to equitable participation, adaptive governance, and knowledge sharing. Mandate regular peer-to-peer learning sessions within the network to promote best practices and strengthen relationships. and ensure outputs are openly shared to foster cumulative knowledge and avoid isolated experiences.

**Address actionable knowledge needs**

Establish ‘Community Innovation Grants’ specifically targeted at supporting local businesses and community organizations to co-develop and test sustainability solutions. Provide free technical assistance and mentorship to ensure they can participate effectively in R&I initiatives and develop clear feedback loops, so community insights demonstrably influence research and innovation agendas.

## Summary and recommendations

### Summary of key challenges of SPPI in Europe

A transition towards sustainability demands a transnational approach, carefully balanced with local tailoring to account for diverse contexts and development pathways both across and within nations. Successfully orchestrating this transition requires inclusive SPPI to foster a shared understanding of priorities, their regional and national implications, and the capacity to translate generated knowledge into actionable solutions within specific local settings.

Our empirical analysis identified several key challenges impacting the effectiveness of SPPI. A primary concern was the fragility and fragmentation of information utilization chains or knowledge-to-action pathways, often compounded by limited resources and capacities. A lack of coordination also restricts opportunities for cross-sectoral and cross-national collaboration. Communication limitations within regulatory bodies were observed, and formalized dialogue and co-creation processes offer significant potential to improve transparency and responsiveness. Reaching and meaningfully engaging a diverse stakeholder base remains a considerable hurdle, particularly given the erosion of trust, increasing societal polarization, nationalism and populism. Moreover, scepticism surrounding scientific evidence, the proliferation of misinformation, and general disengagement from environmental and sustainability concerns pose significant threats. Overcoming these requires deliberate trust-building and consistent, far-sighted and insipiring communication strategies.

Our bottom-up method for identifying and assessing the challenges of SPPI showed that versatile tools are already in use, providing valuable opportunities to learn what works under which circumstances. However, without a systemic approach to learning and adapting these practices, European environmental and sustainability R&I risks remaining a fragmented cacophony of different voices rather than a coordinated engine for societal transformation. Strengthening SPPI in Europe necessitates a coordinated approach encompassing enhanced interdisciplinary and inclusive cross-sectoral collaboration, refined funding mechanisms, targeted capacity building, and accessible communication channels—all of which are essential to ensuring meaningful engagement between science, society, policy and practice.

### Recommendations for strengthening the methods of SPPI

To overcome identified challenges and maximise the long-term societal impact of SPPI, we propose a multi-faceted approach targeting policymakers, researchers, and funders. The use of specific tools or methods of SPPI should be grounded on the following general level principles:

- Foster coordinated and endured collaboration: Policymakers should prioritize the creation and formal support of collaborative networks encompassing government agencies, academic institutions, and third-sector organizations. These networks should be explicitly designed to facilitate knowledge sharing and resource allocation, ensuring their longevity despite policy changes.
- Empower local stakeholders: Broad-based engagement and empowerment of local businesses and communities are vital for ensuring R&I directly addresses local needs and contributes to a just and sustainable transition. Targeted training and resource provision should facilitate meaningful citizen participation. This requires accessible funding, sustained facilitation, involving all parties with explicit inclusion of vulnerable or marginalized groups from the outset.
- Adapt to local contexts: Recognizing distinct cultural, economic and political contexts, SPPI frameworks require adaptation to reflect local realities while retaining alignment with broader EU-level objectives and relevant global discourse. Guidance and toolkits should explicitly address how to navigate these tensions rather than defaulting to a ‘one-size-fits-all’ approach.
- Secure dedicated SPPI funding and robust evaluation: Commit to multi-annual funding cycles, joint budgets for research–policy–practice consortia, and independent evaluation that measures both scientific outputs and societal impacts. Long-term funding commitments and robust evaluation methodologies are essential to ensure project continuity and assess the wide-ranging societal impacts of environmental and sustainability R&I. Joint budgets for research consortia explicitly incorporating interaction activities will ensure a consistent focus on SPPI outcomes. Attracting private sector involvement and investment on environmental and sustainability R&I may alleviate financial burdens on public resources.
- Incentivise collaborative research: Realign academic incentives to actively reward cross-disciplinary, collaborative, and problem-oriented research contributions. This should include financial and societal support for collaborations with non-academic stakeholders from the project’s inception. A particular emphasis should be on developing mechanisms for small and medium sized enterprises related to environmental and sustainability R&I to participate and to test results on their own sites.
- Build societal trust and ensure transparency: Establishing permanent structures to cultivate trust and connect research outcomes with practical application is critical. Transparency means not only disclosing results but also disclosing interests and values. This requires targeted capacity building across all stakeholder groups, underpinned by open and accessible communication and interaction channels.

## Ethics and consent

This study utilizes existing data that were originally collected with verbal informed consent during interviews and workshops. As the data had already been ethically approved and handled, formal ethical review and explicit consent for this secondary analysis were not required. We have adhered to principles of responsible data handling.

## Data Availability

This study uses data from 14 national and regional case studies as reported by Maring *et al.* (2025). The source materials underlying the results are openly available from: http://hdl.handle.net/10138/596051.
