# Supplementary material for: Rethinking science-policy-practice interaction for transformative sustainability research and innovation in Europe
Source: Open Res Eur. 2026 Jan 14;5:351. Originally published 2025 Nov 12. [Version 2] doi: 10.12688/openreseurope.21755.2 (PMC12824481; doi:10.12688/openreseurope.21755.2)
Supplement: Peer review history

# Reviewer report: Reviewer response for version 2

**DOI:** 10.21956/openreseurope.24061.r68071

**Published:** 2026-01-22

**Authors:** Bablu Kumar Dhar

**Affiliations:** 1 Mahidol University, Salaya, Thailand

The revision is a clear improvement in transparency and readability: the authors now state a coherent aim (mapping existing SPPI tools and drawing practical recommendations) and the expanded Background/Methods makes the overall process easier to follow and replicate in principle. The study’s main value is its cross-country synthesis of “what’s being used” and “what gets in the way” (resources, silos, policy context, and transdisciplinarity), and the added limitations section appropriately flags the qualitative, expert-assessment nature of the evidence and the Europe-focused scope. I have only minor remaining suggestions: (i) briefly clarify how “tools/approaches” were identified and consolidated into Table 1 (e.g., coding steps, decision rules, how duplication across countries was handled), (ii) add one or two concrete examples showing how Box 1 steps could be operationalised under different national capacity levels (high-resource vs low-resource contexts), and (iii) tighten a few repeated sentences in the Methods section to avoid redundancy. Overall, I consider the manuscript suitable as a method article and largely ready as-is, with small edits that would further strengthen reproducibility and practical uptake.

Are sufficient details provided to allow replication of the method development and its use by others?

Partly

Is the description of the method technically sound?

Partly

Are the conclusions about the method and its performance adequately supported by the findings presented in the article?

Partly

Is the rationale for developing the new method (or application) clearly explained

Yes

If any results are presented, are all the source data underlying the results available to ensure full reproducibility?

Partly

Reviewer Expertise:

Sustainability

I confirm that I have read this submission and believe that I have an appropriate level of expertise to confirm that it is of an acceptable scientific standard.

# Reviewer report: Reviewer response for version 2

**DOI:** 10.21956/openreseurope.24061.r68183

**Published:** 2026-01-21

**Authors:** Syed Asad Abbas Bokhari

**Affiliations:** 1 Nazarbayev University, Astana, Kazakhstan

**Summary of the Article**

 This method-oriented article examines how science–policy–practice interaction (SPPI) can be strengthened to support transformative sustainability research and innovation across Europe. Drawing on document analysis, semi-structured interviews, and national workshops involving approximately 300 stakeholders across fourteen European countries, the authors map existing SPPI tools and practices and identify systemic challenges that hinder effective knowledge-to-action pathways.

 The study highlights persistent barriers, including fragmented institutional structures, uneven resource allocation, limited transdisciplinarity, and deficits in inclusive participation. Based on comparative synthesis, the authors propose a set of practical and institutional responses, including Sustainability SPPI Forums, contextualization toolkits, innovation networks, coordination funding mechanisms, and principles for inclusive engagement. The revised manuscript clarifies that the core contribution is methodological: it offers a structured, adaptable process for diagnosing SPPI gaps and designing context-sensitive interventions.

 The second version substantially improves clarity and rigor. The aims are now explicit, the methodological workflow is transparent, sampling and participant profiles are clarified through Figure 1, links to open CASRI reports are provided, and a limitations section bounds the scope of claims. The paper now functions convincingly as both an empirical synthesis and a reusable methodological blueprint.

**Review Report**

- The authors have made strong progress toward transparency:
- The data generation process is clearly described.
- Interview schemes, workshop templates, and synthesis procedures are documented.
- Openly accessible CASRI reports are linked.
- The qualitative and comparative nature of the analysis is explicitly acknowledged.

However, full reproducibility in the strict sense is not possible because:

- The primary materials consist of qualitative interviews and workshops conducted in national languages.
- Raw transcripts and workshop records are not publicly available.
- The synthesis relies on interpretive and consensus-based qualitative analysis across national teams.

This is acceptable and common for large-scale qualitative and comparative studies. The revised manuscript now enables methodological replication even if data replication is not feasible. Another research team could reproduce the process in a different context using the same logic, instruments, and workflow.

**Scientific Soundness**

 All issues that previously affected methodological clarity and reproducibility have been addressed:

- The purpose of the study is now unambiguous.
- Sampling and participant composition are transparent.
- The analytical workflow is clearly described.
- External documentation is openly linked.
- Limitations and scope conditions are explicit.

There are no remaining points that must be addressed to make the article scientifically sound. The manuscript now meets the standards for a method article in terms of rigor, transparency, and contribution.

**Final Recommendations:**

 The revised manuscript demonstrates a clear and thoughtful response to all substantive concerns raised in the previous review round. The authors have significantly strengthened the paper’s methodological transparency, conceptual clarity, and practical relevance.

 In particular:

- The aims of the study are now articulated with precision, explicitly positioning the paper as a methodological and analytical blueprint for mapping and strengthening science–policy–practice interaction (SPPI).
- The Background and Methods section has been substantially expanded and now provides a coherent, step-by-step account of data generation, stakeholder recruitment, and analytical synthesis, including the introduction of Figure 1 to clarify the composition of participants.
- The authors have improved reproducibility by linking to openly accessible project reports and by detailing interview schemes, workshop structures, and the iterative synthesis process.
- A dedicated limitations discussion now acknowledges the qualitative and European scope of the study, enhancing both rigor and transparency.
- The pathway from empirical challenges to the proposed SPPI recommendations is clearer and more defensible, with explicit criteria and grounding in the collected evidence.

These revisions directly address the reviewer’s concerns regarding methodological rigor, transferability, and transparency. The manuscript now functions convincingly as a method article that others can adapt in different regional and institutional contexts. The paper offers both conceptual value and concrete, operational guidance for scholars, policymakers, and research funders engaged in sustainability transitions.

 The revised version meets the standards for clarity, rigor, and contribution. I therefore recommend this manuscript for acceptance without further revision.

Are sufficient details provided to allow replication of the method development and its use by others?

Yes

Is the description of the method technically sound?

Yes

Are the conclusions about the method and its performance adequately supported by the findings presented in the article?

Yes

Is the rationale for developing the new method (or application) clearly explained

Yes

If any results are presented, are all the source data underlying the results available to ensure full reproducibility?

Partly

Reviewer Expertise:

AI Applications in governance, sustainability, environment... Environmental sustainability, Technological innovation

I confirm that I have read this submission and believe that I have an appropriate level of expertise to confirm that it is of an acceptable scientific standard.

# Reviewer report: Reviewer response for version 2

**DOI:** 10.21956/openreseurope.24061.r68072

**Published:** 2026-01-16

**Authors:** Aktham Maghyereh

**Affiliations:** 1 United Arab Emirates University, Al Ain, United Arab Emirates

The authors have revised the manuscript and have appropriately addressed all comments and suggestions raised in the previous review. The revisions have improved the clarity and quality of the paper, and I have no further concerns.

Are sufficient details provided to allow replication of the method development and its use by others?

Partly

Is the description of the method technically sound?

Yes

Are the conclusions about the method and its performance adequately supported by the findings presented in the article?

Partly

Is the rationale for developing the new method (or application) clearly explained

Yes

If any results are presented, are all the source data underlying the results available to ensure full reproducibility?

No source data required

Reviewer Expertise:

Sustainability

I confirm that I have read this submission and believe that I have an appropriate level of expertise to confirm that it is of an acceptable scientific standard.

## References

[1] : Rethinking science-policy-practice interaction for transformative sustainability research and innovation in Europe. *Open Research Europe* .2026;5: 10.12688/openreseurope.21755.2 10.12688/openreseurope.21755.2 PMC1282448141586437

# Reviewer report: Reviewer response for version 1

**DOI:** 10.21956/openreseurope.23534.r64145

**Published:** 2025-12-01

**Authors:** Aktham Maghyereh

**Affiliations:** 1 United Arab Emirates University, Al Ain, United Arab Emirates

The article proposes a structured approach for **rethinking science-policy-practice interaction (SPPI)** to support transformative sustainability research and innovation in Europe. The authors combine insights from a large set of workshops and activities (~300 participants across 20+ European countries) with a “funnelling” analytical process to identify key challenges, gaps, and opportunities in SPPI for sustainability transitions.

 They first review existing tools and approaches for SPPI (e.g., information-sharing tools, co-design workshops, science advisory bodies, campaigns and events), and then synthesize workshop results into a set of core European challenges such as resource constraints, siloed institutional structures, limited transformative transdisciplinarity, and inclusivity deficits. On this basis, they develop a set of **applied recommendations**, including institutionalised “Sustainability SPPI Forums”, contextualisation toolkits, SPPI innovation networks, and dedicated coordination funds to strengthen cross-sectoral collaboration and transformative capacity. 

 Comments: 

 1. The authors might add 1–2 sentences explicitly framing the contribution as a *methodological blueprint* (or “process model”) that other regions or programmes can adapt, to make the methodological added value even more explicit.

 3. While the manuscript provides approximate numbers of participants and participating countries, the description of *sampling and coverage* would benefit from greater specificity, particularly regarding how workshops were selected, how stakeholders were recruited, and which types of organisations (academia, ministries, NGOs, industry, local authorities, etc.) were represented or under-represented. In addition, the *analytical procedure* requires clearer methodological transparency: although the paper notes that workshop outputs were “compiled into internal comprehensive reviews and synthesised into a single, openly available report,” a concise, step-by-step account of how data were coded, clustered, and synthesised (e.g., thematic analysis, iterative coding cycles, inter-author validation) would significantly improve reproducibility. Finally, while the proposed SPPI interventions—such as Sustainability SPPI Forums, the Contextualisation Toolkit, SPPI Innovation Networks, and the Coordination Fund—are well-articulated, the *criteria for moving from identified challenges to these specific recommendations* should be more explicitly described, including considerations such as feasibility, scalability, institutional suitability, and equity. This additional detail would help readers understand the rationale behind the design choices and strengthen the methodological contribution of the paper.

 3. Consider providing, as supplementary material or in a repository, (i) sample workshop agendas, (ii) data extraction templates, and (iii) a schematic of the coding/synthesis workflow. This would significantly enhance reusability of the method.

 4. For strict replication of the method, the manuscript would benefit from additional operational detail. Specifically, providing examples of the workshop guides, question prompts, or facilitation protocols used to elicit SPPI challenges and opportunities would greatly clarify how the sessions were structured. A brief description of how participant feedback was recorded and aggregated—whether through written notes, online collaboration tools, standardized templates, or other documentation procedures—would also enhance transparency. Furthermore, it would be helpful to explain how divergent or conflicting views across countries were managed during the synthesis stage, including whether disagreements were reconciled through consensus building, weighting procedures, or iterative validation among authors. Incorporating these details would substantially strengthen the reproducibility and methodological rigor of the study.

 5. It would be helpful to clearly link to that report (and, if possible, to annexes or summaries from the workshops) and to specify what materials are publicly accessible versus internal. This is especially relevant because the paper is positioned as a method article.

 6. To strengthen the link between the method’s performance and the conclusions drawn, the manuscript would benefit from a more explicit reflection on what aspects of the process worked effectively and which proved more challenging. For instance, identifying which SPPI tools or workshop formats generated the most meaningful insights, where participation remained narrow or uneven, or where translation of workshop outputs into policy recommendations was difficult would help readers better understand the practical strengths and limitations of the approach. Additionally, incorporating a brief subsection on limitations and transferability, discussing the extent to which the findings are context (dependent and which components of the method may require adaptation in non-European or less-resourced settings) would add nuance and broaden the relevance of the study. Such reflections would more clearly articulate the method’s applicability and boundaries, thereby enhancing both its credibility and utility for future SPPI initiatives.

Are sufficient details provided to allow replication of the method development and its use by others?

Partly

Is the description of the method technically sound?

Yes

Are the conclusions about the method and its performance adequately supported by the findings presented in the article?

Partly

Is the rationale for developing the new method (or application) clearly explained

Yes

If any results are presented, are all the source data underlying the results available to ensure full reproducibility?

No source data required

Reviewer Expertise:

Sustainability

I confirm that I have read this submission and believe that I have an appropriate level of expertise to confirm that it is of an acceptable scientific standard, however I have significant reservations, as outlined above.

# Response

**Published:** 2025-12-15

**Authors:** Jari Lyytimäki

**Affiliations:** Finnish Environment Institute, Helsinki, Uusimaa, Finland

Thank you for the insightful and constructive review. We have revised the manuscript based on the advice. The revisions include several additions and clarifications addressing the review comments.

 Furthermore, some overlapping or wordy expressions have been removed or shortened to keep the manuscript concise. Please find point-by-point responses below. 

**1. Response: **As rightly pointed out, the aim of the study should have been more clearly stated. We have reformulated the aims statement in the second-to-last paragraph of the introduction, clearly stating our focus on mapping the tools and methods of SPPI and making recommendations based on the analysis. 

**2. Response: **More detailed information on the sampling strategy, recruitment, and background of the interviewees is now provided, including the new Fig. 1 summarizing the roles, organizational background, and expertise of the respondents. A description of the steps of data generation and analysis has been added. Links to openly accessible reports providing detailed descriptions of the research process are also provided to enhance transparency. It is now declared that our analysis relied on qualitative expert assessment, which was considered suitable for a comparative case study approach integrating a variety of different national contexts. The criteria for moving from identified challenges to recommendations are also outlined at the end of the section on Background and Methods, emphasizing the long-term societal impact of SPPI, the ability to address multifaceted challenges, and the effectiveness of targeting and engaging diverse stakeholder groups as key criteria. 

**3. Response: **Concise descriptions of the organization of data collection and processing are now added to the text. Links to the open-access report appendices presenting the detailed workshop agendas/structures and semi-structured interview scheme are provided. Our reliance on qualitative interpretations as a method to synthesize insights from different cases is clearly stated as part of the comparative case study approach. 

**4. Response: **In order to ensure the transparency and replicability of the research process, essential elements of the interview scheme and descriptions of desktop studies and workshops are now added. References to the rather lengthy and detailed documentation are provided. Our synthesis relied on qualitative interpretations and iterative consensus building and validation among authors. This is now outlined at the end of the section describing the Background and Methods. 

**5. Response: **References to the open-access reports (with annexes) outlining the methods for data generation and presenting the results serving as a basis of this analysis are now provided more carefully. 

**6. Response: **As we have now clarified, the main aim of this manuscript was not to develop a new method for assessing SPPI but to map the use of SPPI tools and methods and make recommendations from the perspective of sustainability transition. A paragraph focusing specifically on the limitations of this study is added to the end of the Background and Methods section. We also highlight that the focus here is on European experiences. Even though we hope that our recommendations are widely applicable, we feel that evaluating their usefulness beyond European contexts is beyond the remit of our current manuscript.

# Reviewer report: Reviewer response for version 1

**DOI:** 10.21956/openreseurope.23534.r64142

**Published:** 2025-11-27

**Authors:** Bablu Kumar Dhar

**Affiliations:** 1 Mahidol University, Salaya, Thailand

The manuscript offers rich empirical insights, but to fully meet the expectations of a Method Article the methodological contribution needs to be made more explicit and operational. I encourage the authors to clearly articulate in the introduction and Background and methods sections how their approach improves upon or differs from existing SPPI and transdisciplinary methods (e.g. in terms of scope, scale, governance and the funnelling approach), and to consider adding a visual representation of the methodological steps and data flows. For replication, the methods section would benefit from more operational detail, including the sampling strategy for interviewees and workshop participants (with sectoral and, where possible, gender considerations), the core elements of the interview guide and workshop design, and a fuller description of the qualitative content analysis and cross-country synthesis; if space is limited, these could be provided in an appendix or supplementary material. Transparency could be strengthened by clarifying the status and handling of the underlying data (interviews, workshop notes, national reports), how they are stored and accessed, and how country-level findings were aggregated (e.g. use of a common coding framework and whether multiple authors cross-checked interpretations). A short, explicit subsection on limitations would further enhance robustness, for instance by acknowledging potential biases in expert selection, uneven sectoral coverage, differences in national implementation, language issues, and the fact that the work mainly reflects high-income European contexts. Finally, in the Summary and Recommendations, it would be helpful to distinguish more clearly between (a) empirical conclusions about the state and challenges of SPPI in Europe and (b) methodological conclusions about the proposed approach to assessing and improving SPPI, including its strengths, constraints, and recommended conditions for future use.

Are sufficient details provided to allow replication of the method development and its use by others?

Partly

Is the description of the method technically sound?

Partly

Are the conclusions about the method and its performance adequately supported by the findings presented in the article?

Partly

Is the rationale for developing the new method (or application) clearly explained

Yes

If any results are presented, are all the source data underlying the results available to ensure full reproducibility?

Partly

Reviewer Expertise:

Sustainability

I confirm that I have read this submission and believe that I have an appropriate level of expertise to confirm that it is of an acceptable scientific standard, however I have significant reservations, as outlined above.

# Response

**Published:** 2025-12-15

**Authors:** Jari Lyytimäki

**Affiliations:** Finnish Environment Institute, Helsinki, Uusimaa, Finland

Thank you for the insightful and constructive review. We have revised the manuscript and made the following key changes.

 The focus on reviewing SPPI methods and its aim is now clearly stated in the introduction. The section on Background and Methods has been completely rewritten, and a more detailed description of the research process has been added. Key phases of stakeholder involvement and analysis are described, taking into account space limitations. The criteria and process for identifying interviewees are now detailed. The reference to the ‘funnelling approach’ has been removed, as it referred to the overall project approach and was designed primarily to guide the selection of environmental and sustainability R&I topics rather than support data collection specifically focusing on SPPI. Therefore, we considered that mentioning it was not essential here. A figure summarizing the background information of the involved expert stakeholders has been added. The detailed interview scheme and guidance for workshop organization are openly available as appendices in Maring et al. (2025). This is now clearly noted, and the essential elements are briefly described. Transparency has been improved by describing the data generation process in more detail. It is also mentioned that this research does not analyze the underlying national-level data but uses the already reported transnational results as a starting point. A paragraph on limitations has been added at the end of the section on background and methods. Finally, empirical conclusions focusing on Europe and methodological recommendations focusing on guiding principles of SPPI are now clearly distinguished in the Summary and Recommendations section.

 Furthermore, we clarified that Box 1 presents our proposal on the development of SPPI methods, summarizing practice-oriented steps that serve as an accessible and actionable blueprint adaptable to various communication contexts. We hope that this clarifies that the main aim here is to map the use of SPPI tools and methods in Europe and support their development, rather than develop a new method for assessing SPPI.
